# Construction of Custom Ocular Prosthesis With Reduced Number of Visits: A Case Report

**DOI:** 10.7759/cureus.48159

**Published:** 2023-11-02

**Authors:** Mohammad Majduddin Sulaiman, Nor Aidaniza Abdul Muttlib, Rabihah Alawi

**Affiliations:** 1 Prosthodontics Unit, School of Dental Sciences, Health Campus, Universiti Sains Malaysia, Kelantan, MYS; 2 Prosthodontics Unit, Hospital Universiti Sains Malaysia, Kelantan, MYS; 3 Unit of Conservative Dentistry, School of Dental Sciences, Health Campus, Universiti Sains Malaysia, Kelantan, MYS; 4 Unit of Conservative Dentistry, Hospital Universiti Sains Malaysia, Kelantan, MYS

**Keywords:** prosthodontic, maxillofacial prosthesis, dental visits, ocular defect, ocular prosthesis

## Abstract

Construction of an ocular or eye prosthesis can be challenging, as it is a cosmetic device. It needs good communication between dentists and maxillofacial technicians. The construction process normally requires multiple appointments, which involve at least four visits starting with ocular impression to insertion of the prosthesis. This article will outline the clinical step and propose a clinical technique to reduce the number of appointments from four to three appointments, which would benefit both the patient and practitioner.

## Introduction

The eye plays a crucial role in humans; the role ranges from vision to emotional expression. The loss of an eye can bring physical, social, and psychological impacts on those who are affected. The removal of an eye or eye loss is due to either trauma or pathological or congenital causes [1]. The surgical procedures for eye removal include evisceration, enucleation, and exenteration.

Evisceration can be defined as the removal of ocular contents through a corneal or scleral incision while preserving the conjunctiva, sclera, extraocular muscles, and orbital fat. Meanwhile, enucleation is the complete removal of an eyeball following the disinsertion of extraocular muscles and optic nerve, and exenteration is where the whole content of the orbit, including the eyelids and surrounding tissues, are removed [2].

When the surgical site heals and is dimensionally stable, the ocular prosthesis can be planned and constructed. Early management of ophthalmic sockets will prevent the loss of orbital volume and subsequently facial asymmetry [3]. Besides, it will improve the quality of life of the patients.

There are two types of ocular prostheses which are ready-made (stock) and custom-made. Compared to a ready-made prosthesis, a custom ocular prosthesis has better adaptation to the tissue bed for the patient’s comfort and has the potential to produce eye movement that looks more natural [4]. It also eliminates voids around fitting surfaces that can irritate mucosa which can be infected.

In general, the conventional prosthetic process involves accurate impression taking, wax try-in, iris positioning, eye painting, and fitting of the prosthesis. It requires at least four clinical visits by the patient in the fabrication process, excluding review visits. As there are many clinical techniques for ocular prosthesis fabrication, this paper aimed to highlight the technique used in our setting while considering how to reduce the number of appointments by patients.

## Case presentation

A 22-year-old male patient was referred to the prosthodontic clinic. He requested a left eye prosthesis due to his existing prosthesis being broken. The history revealed that he had left phthisis bulbi secondary to a motor vehicle accident. He underwent left eye enucleation with an orbital implant three years ago. After the healing was achieved, he was issued with a left eye prosthesis. However, with time, he noticed that the eye prosthesis was loose and easily dislodged from the socket.

**Figure 1:**
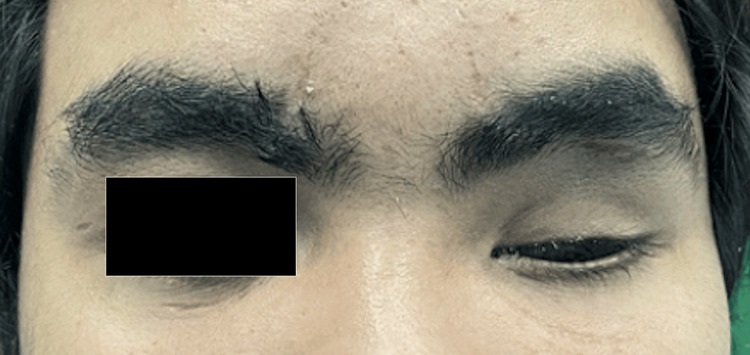
Pre-treatment; the patient with a left ocular defect

On clinical examination, there was ptosis of the left eyelid with healthy conjunctiva and there was no sign of infection (Figure 1). The ocular implant was intact on palpation. The left eye sulcus was adequate to retain the new prosthesis. The author could not examine the existing prosthesis since the patient reported he had discarded the prosthesis. After consultation and discussion, the treatment plan was decided to construct a new prosthesis. Written informed consent was obtained.

**Figure 2:**
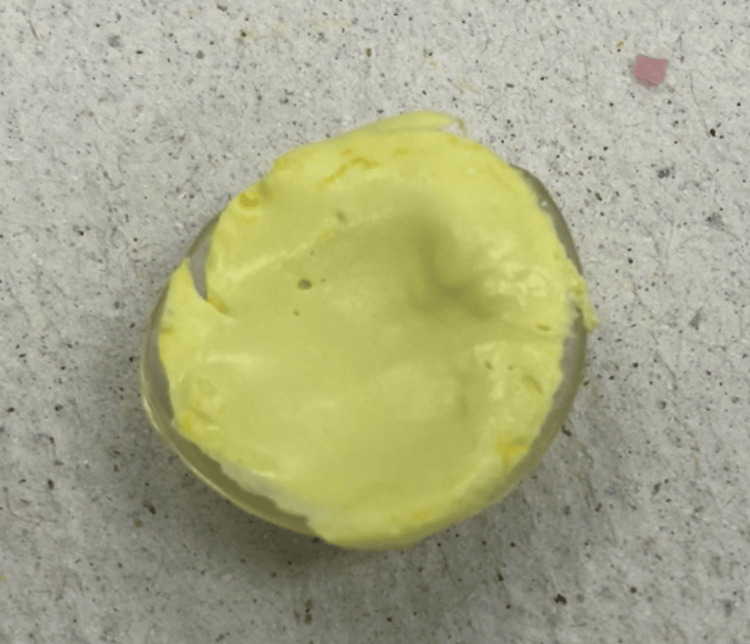
Ocular impression on the stock tray

Petroleum jelly was applied to the eyelash and surrounding surface of the left orbit to prevent the impression from sticking to the tissue. Primary impression was recorded using heavy body polyvinyl siloxane impression material (Examix NDS, Japan) and a stock tray fabricated by our technician. During the procedure, the patient was asked to perform functional eye movement, including medial, lateral, superior, inferior, and rotational movements. This was to ensure that the impression material would record all the anatomical details of the ophthalmic socket. Then, the patient was asked to stare straight ahead until the impression was set to ensure the tissue bed was recorded at rest (Figure 2).

**Figure 3:**
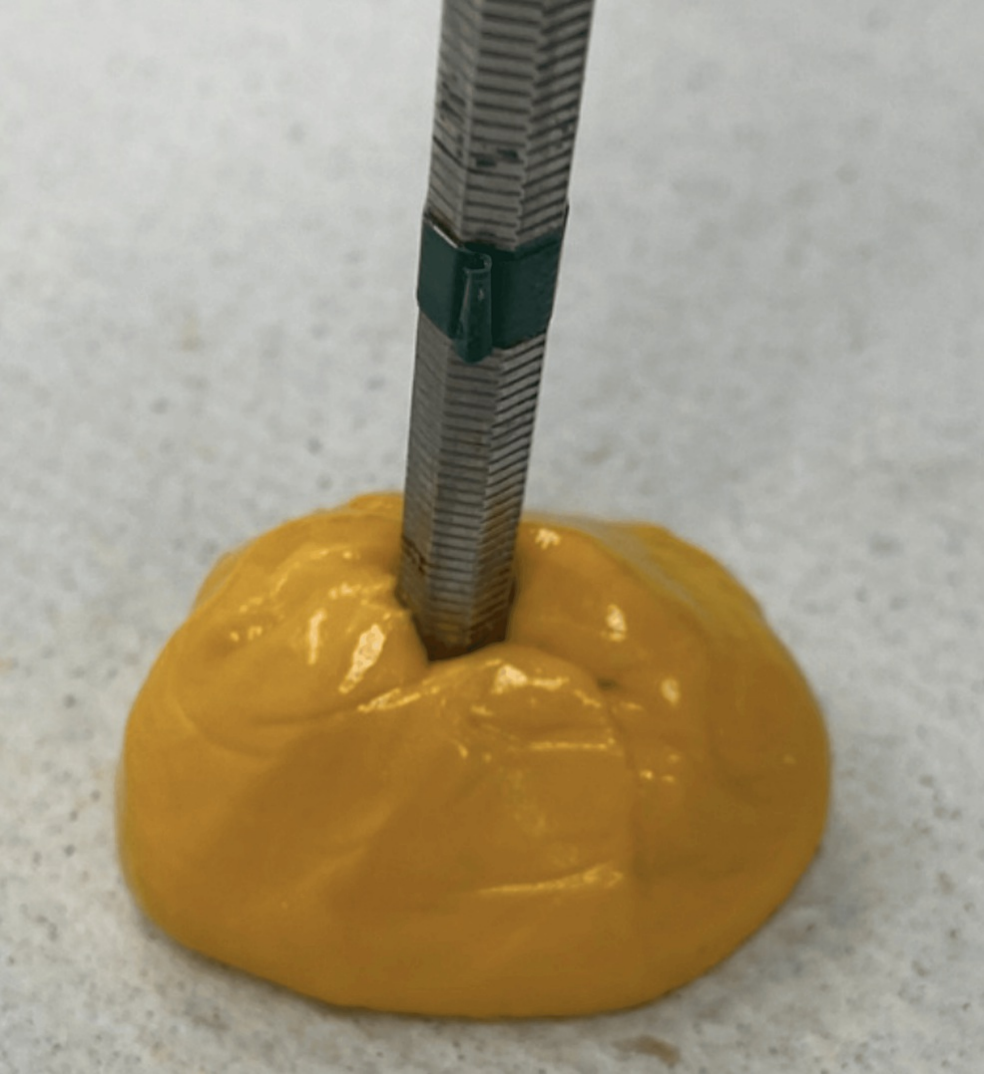
Mold construction with the dental mirror handle as a sprue

The mold for the wax pattern was created using putty polyvinyl siloxane (3M Deutschland GmbH, Germany). A mirror handle was used on the center to create a sprue (Figure 3).

**Figure 4:**
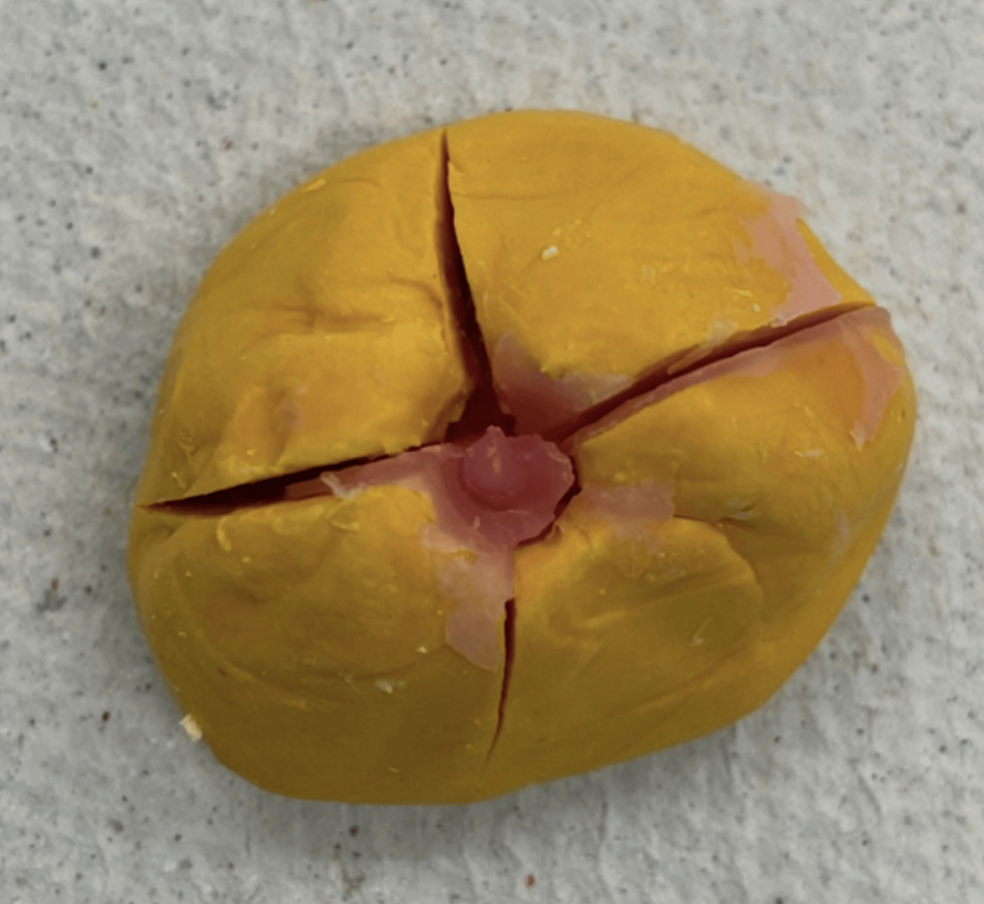
Wax pattern construction using a mold

**Figure 5:**
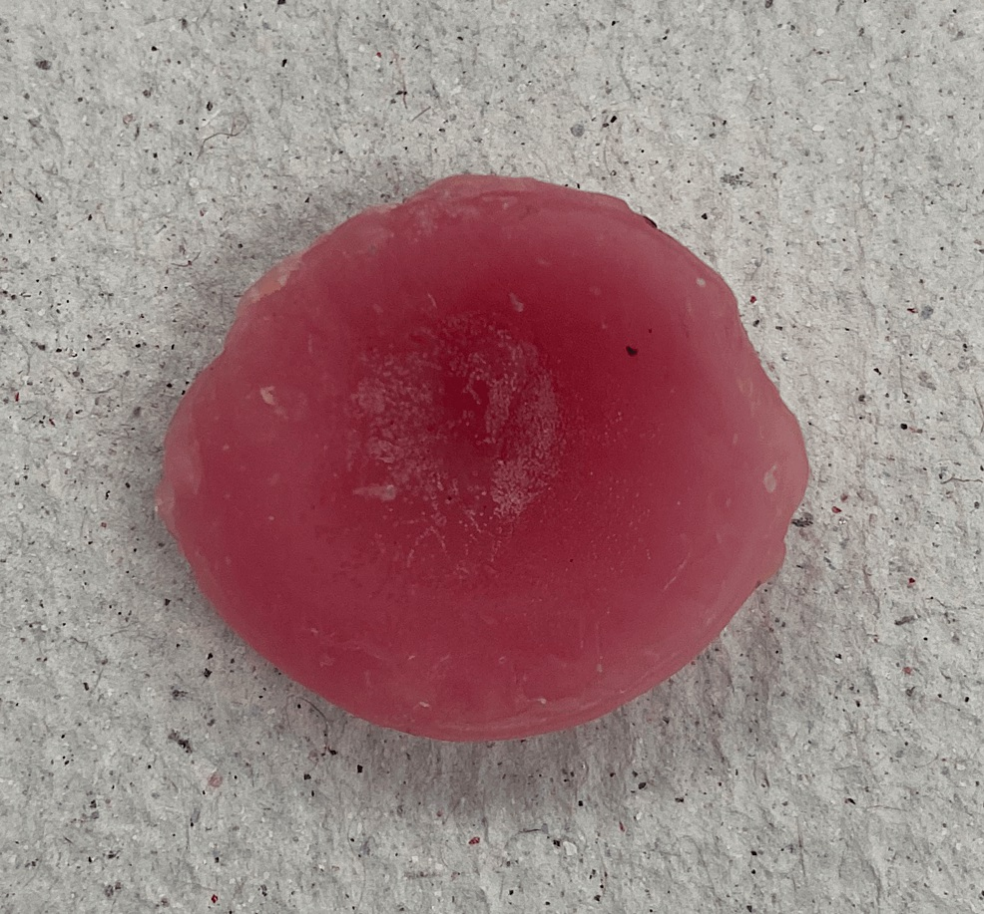
Ocular wax pattern

Once the putty set, the impression was removed from the mold and melted wax was poured through the sprue to construct a wax pattern (Figure 4). Any sharp fins on the wax pattern were smoothened using a wax knife (Figure 5).

**Figure 6:**
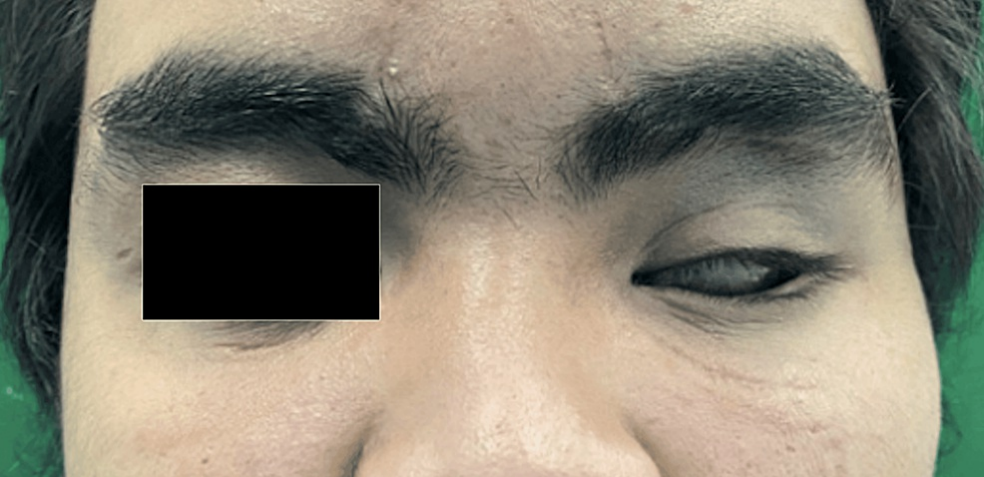
Try-in of ocular wax pattern.

The ocular wax pattern was tried into the eye socket and carved accordingly (Figure 6). The size, retention, motility, and fullness were checked. The patient was instructed to sit upright and look straight. In this position, the iris size was selected and marked on the wax pattern by comparing the contralateral eye as a guide.

**Figure 7:**
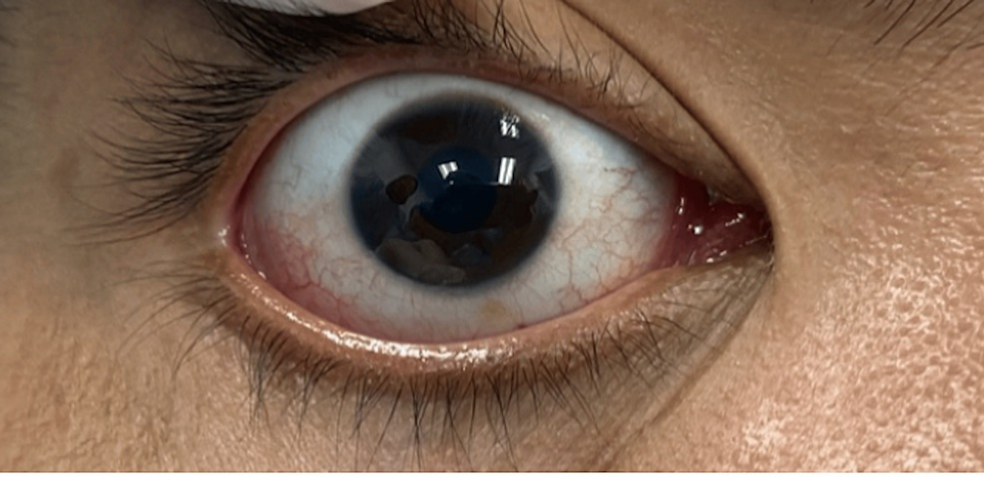
Digital photo of the contralateral eye as a guide for the technician to color the iris and eye

A digital photograph of the contralateral right eye and iris was made to facilitate the coloring by the technician (Figure 7). The patient was discharged after this procedure. The wax pattern was sent to the technician for iris placement.

**Figure 8:**
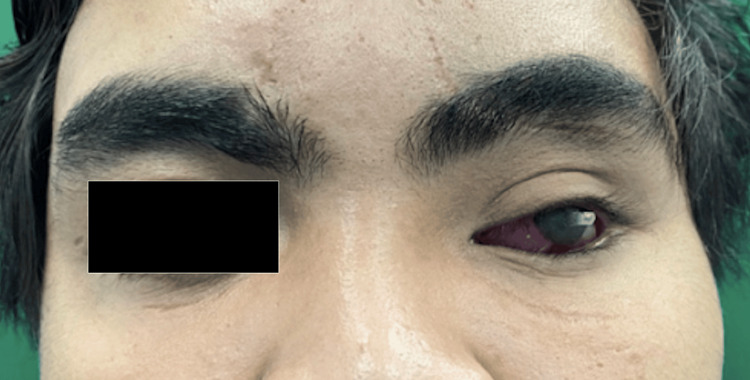
Try-in of the ocular wax pattern with the iris and pupil button

During the second appointment, a try-in of the wax pattern with the iris was done (Figure 8). During this visit, the shape, iris position, and color were finalized. Once the patient approved the try-in of the prosthesis, the wax pattern was then sent for flasking, processing, finishing, and polishing. This step was the end of the second visit for the patient.

**Figure 9:**
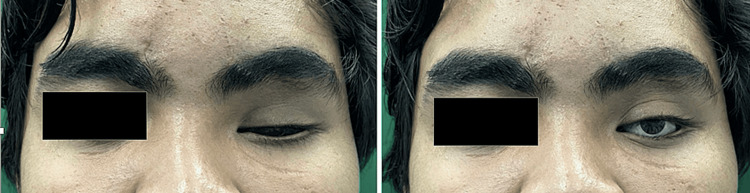
Before (left) and after (right) fitting the left eye prosthesis

The left eye prosthesis was inserted and given to the patient on the next visit (Figure 9). The patient was asked about any pain or discomfort and the problematic area was trimmed with acrylic bur. Instructions regarding the maintenance and hygiene of the ocular prosthesis were provided.

A review appointment was scheduled at one-week post-insertion. He had no complaints and was comfortable with the new eye prosthesis. A recall appointment was given one month and one-year post-insertion.

## Discussion

Over the years of wearing a prosthesis, it is common for the prosthesis to become loose due to atrophy of fat and muscle. Other causes include the growing child or the prosthesis being constructed too soon before the edema subsides [5]. In this patient, the potential cause may be due to fat and muscle atrophy, which change the ophthalmic socket leading to poor fit and loss of retention. A one-year recall is recommended for all eye prosthesis patients to monitor any changes and proper intervention can be carried out [5,6].

Several techniques have been discussed in fabricating ocular prostheses, which include modifying stock eye or custom-made techniques. The construction of custom-made eye prostheses is seen as superior to the stock eye, which has a better aesthetic and is more precise [4]. A conventional technique for ocular prosthesis construction involves at least four visits excluding a review visit, which includes impression making on the first visit, a try-in wax pattern and positioning of the iris on the second visit, try-in iris on the third visit, and the insertion on the fourth visit [7].

In this report, the author proposes techniques to minimize the number of visits by patients in custom-made eye prostheses by combining the first and second visits together. The first visit includes examination and diagnosis, treatment planning, impression, wax pattern try-in, and iris marking. All the steps described above were done by the author at the chair side, which also minimizes impression errors. The second visit was an ocular wax pattern try-in with the iris and the third visit was when the eye prosthesis was inserted. Although these techniques lengthen the time spent on the first visit, the number of visits attended by patients was decreased by one session, which simultaneously reduced the cost such as traveling costs by patients. The prosthesis made with these fewer clinical visits is able to produce functional and aesthetic results that are comparable to those of the conventional method.

This technique is limited to patients with adequate eye sulcus and dimensionally stable eye sockets. For unstable eye sockets such as after the surgery, the conformer will be issued for the patient to wear. The conformer acts as an interim prosthesis to minimize changes in socket size and conformation and prevent scar tissue contraction, thus facilitating the fabrication of a definitive prosthesis [8]. While treating this patient, no conformer is being issued prior to a definitive prosthesis in view of his socket being dimensionally stable.

The patient presented with ptosis, which has been described as one of the complications of eye enucleation. Other complications include enophthalmos, deepening of the superior sulcus, backward tilting of the prosthesis, and stretching of the lower eyelid. The symptoms are described as post-enucleation socket syndrome, which may exist separately or in combination with varying degrees of severity [9]. The orbital loss should be replaced with an adequate volume of the orbital implant to prevent or minimize complications [10].

Every patient who receives the prosthesis should be reinforced on ocular prosthesis care instructions. These care instructions include that the patients will be asked to clean the prosthesis with mild soap every one to two weeks, to remove the ocular prosthesis only when necessary, and if removed, to store in water or contact lens saline solution to avoid drying [5,6].

## Conclusions

Custom-made ocular prosthesis fabrication with fewer and more effective visits was presented in this case report as an alternative to the conventional technique. It eventually reduces the cost of attending appointments with the patient. The ocular prosthesis also has similar functional and aesthetic outcomes with conventional techniques. It is based on the clinician's judgment to use this technique with limitations as described above.
